# Studies of UPLC fingerprint for the identification of *Magnoliae officinalis* cortex processed

**DOI:** 10.4103/0973-1296.62891

**Published:** 2010-05-05

**Authors:** Lin Wang, Ke Yuan, Wei-wu Yu

**Affiliations:** 1 *Research and Development Center of Natural Medicine, Zhejiang Forestry University, Lin'an 311300, P. R., China*; 2 *College of Pharmacy, Henan University of Traditional Chinese Medicine, Zhengzhou - 450 008, P.R, China*; 3 *Zhejiang Provincial Key Lab for Modern Silvicultural Technology, Lin'an - 311 300, P. R. China*

**Keywords:** Fingerprint, *Magnoliae officinalis* cortex, Ultra Performance Liquid Chromatograph

## Abstract

This study was carried out with the objective of establishing Ultra Performance Liquid Chromatograph (UPLC) fingerprint for the identification of *Magnoliae officinalis* cortex processed. It was extracted by methanol using an ultrasonic extractor. Twelve samples of *M. officinalis* cortex produced in Zhejiang of China from different places and species were processed with ginger juice; sample solutions were determined by Waters UPLC equipped with BEH C_18_ column and a DAD detector, gradient eluted with formic acid/methanol-formic acid/water as mobile phase. The flow rate was set at 0.3 ml^•^ min^−1^, while the column temperature was set at 30°C, and the wavelength for detection was set at 240 nm. The characteristic of the common peaks of the UPLC fingerprint for *M. officinalis* cortex processed are obvious. Forty-one common peaks were detected and two of them were identified. The method of UPLC fingerprint established in this experiment was rapid and efficient. It is an effective means for the quality control of *M. officinalis* cortex processed.

## INTRODUCTION

Nowadays, Traditional Chinese Medicine (TCM) is more and more popular and widely used clinically for its excellent qualities such as low toxicity and less side effects, good medical effects and rare drug tolerance. It has long been proved that the major difference between TCM and chemical medicine is that TCM cures diseases by multiple components and multiple target points. However, the traditional HPLC fingerprints cannot meet the requirements of high throughput analysis and greatly restrict the actual use of liquid chromatograph fingerprint in quality control, because the efficiency of column in the joint liquid chromatograph is low and its separating time is long, generally more than 1 h.[1–3] The Ultra Performance Liquid Chromatograph (UPLC) can solve this problem. Compared with the traditional HPLC method, it has the unparalleled advantages of ultra-high column efficiency, separation capability and separation velocity. Under the similar or better separating effect, it can greatly reduce the operating time.[4–10] Therefore, the UPLC fingerprint technology has a vast development prospect and will certainly be used more and more widely as an ideal means of quality control of the Chinese medicine.

*Magnoliae officinalis* cortex belongs to the dried bark of stem, root and branches in *Magnolia officinais* Rehd.et Wils or *Magnolia officinalis* Rehd. et Wils. var. biloba Rehd. et Wils.of *Magnoliaceae* family. It is the commonly used traditional Chinese medicine, and is included in Chinese Pharmacopoeia of 2005 edition.[11] *Magnoliae officinalis* cortex product is the staple dominant medicine herb, and it is a main source of *Magnoliae officinalis* cortex included in Chinese Pharmacopoeia.

Zhejiang province has a large planting area of high quality, with *Magnoliae officinalis* cortex as one of its main medicine producing areas. Every year, a large amount of *Magnoliae officinalis* cortex is used clinically and is exported to south Asia and many other countries and various areas. Production and processing of *Magnoliae officinalis* cortex have become one of the main businesses for the medicine farmers in Zhejiang province.

At present, the processed methods of *Magnoliae officinalis* cortex lack the quantitative criterion, which result in the varying qualities. Through the technology of UPLC fingerprints, we can establish the best fingerprints of *Magnoliae officinalis* cortex processed sample, and quickly and reliably evaluate its inner quality, and at the same time, provide the reference for its clinical use. The literature searches show that nobody ever uses UPLC fingerprints determination technology for the quality evaluation of *Magnoliae officinalis* cortex processed sample. This research established the determination technology and method for UPLC fingerprints of *Magnoliae officinalis* cortex processed sample, and thus provides the reference for the all-round objective evaluation of the quality of *Magnoliae officinalis* cortex processed sample.

## MATERIALS AND METHODS

### Instruments

A Waters Acquity UPLC™ system (Waters Company., MA, USA), with a binary solvent manager, an automatic samplefeeding device and DAD binary lineup examiner were used for liquid chromatographic analysis. Shumei KQ2200DE ultrasonic cleaning instrument (Kunshan Shumei Ultrasonic Instrument Co., Kunshan, China) was used for extraction. AUTO SCIENCE solvent filtration device, Senxin DGG-9240A type drying oven, Milli-Q super-purified water device of Millipore company (Bedford, MA, USA) were the other instruments used.

### Reagents and materials

Magnolol and hononkiol standards were purchased from National Institute for the Control of Pharmaceutical and Biological Products (Peking, China). The batch numbers were 110729-200310 and 110730-200307, respectively, the purity was more than 98%.

The twelve samples from two species (*Magnolia officinalis* Rehd.et Wils. And *Magnolia officinalis* Rehd. et Wils. var. *biloba* Rehd. et Wils.) were collected from Longquan, Jingning, Tiantai, Suichang and Yunhe countries in Zhejiang Province, China, and all were identified as the dried bark of stem, root and branches of *Magnolia officinalis* Rehd.et Wils. or *Magnolia officinalis* Rehd. et Wils. var. *biloba* Rehd. et Wils. by Professor Lou Lu-huan of Zhejiang Forestry University [Table 1]. Then the samples were processed, pulverized and dried according to the method stipulated in Chinese Pharmacopoeia.[7] All the voucher specimens were deposited in our laboratory.

**Table 1:** Representative samples of *Magnoliae officinalis* cortex investigated in this study

Sample no.	Herbal species	Collection site	Collection time
S1	*Magnolia officinalis* Rehd. et Wils. var. biloba Rehd. et Wils.	Tian Tai Country, Zhejiang province	2009-04-16
S2	*Magnolia officinalis* Rehd. et Wils. var. biloba Rehd. et Wils.	Tian Tai Country, Zhejiang province	2009-04-16
S3	*Magnolia officinalis* Rehd. et Wils. var. biloba Rehd. et Wils.	Jing Ning Country, Zhejiang province	2009-04-18
S4	*Magnolia officinalis* Rehd. et Wils. var. biloba Rehd. et Wils.	Jing Ning Country, Zhejiang province	2009-04-18
S5	*Magnolia officinalis* Rehd. et Wils. var. biloba Rehd. et Wils.	Jing Ning Country, Zhejiang province	2009-04-18
S6	*Magnolia officinais* Rehd.et Wils.	Jing Ning Country, Zhejiang province	2009-04-19
S7	*Magnolia officinais* Rehd.et Wils.	Long Quan Country, Zhejiang province	2009-04-27
S8	*Magnolia officinais* Rehd.et Wils.	Long Quan Country, Zhejiang province	2009-04-27
S9	*Magnolia officinais* Rehd.et Wils.	Long Quan Country, Zhejiang province	2009-04-30
S10	*Magnolia officinais* Rehd.et Wils.	Yun He Country, Zhejiang province	2009-05-03
S11	*Magnolia officinalis* Rehd. et Wils. var. biloba Rehd. et Wils.	Sui Chang Country, Zhejiang province	2009-05-10
S12	*Magnolia officinalis* Rehd. et Wils. var. biloba Rehd. et Wils.	Sui Chang Country, Zhejiang province	2009-05-12

*Magnoliae officinalis* cortex processed samples with 12 different producing areas were shredded for process by the determined best processing method. It involves the addition of 5% ginger juice and further cooking by moistening after adding doubled amount of water till it becomes dry. Then it was kept in oven for drying at 55° and then smashed to powder form, and then sifted through the 50-mesh sieve for later use.

The HPLC grade methanol was obtained from American Tedia company, HPLC grade formic acid was from German Merk company, and ultrapure water was self-made by super water purification system. All the other reagents used in sample preparation were of analytical reagent (AR) grade.

### UPLC Chromatographic conditions

#### Chromatograph column

Sample solutions were determined by Waters acquity BEH C_18_ column (50 mm×2.1 mm,1.7 μm, Waters company). The mobile phase consisted of (A) formic acid/methanol (0.2:100, v/v) and (B) formic acid/water (0.1:100, v/v).

The most suitable linear gradient elution program can be seen in Table 2. The flow rate is 0.3 ml/min, and the temperatures of the chromatographic column and the automatic sample-feeding room are 30° and 4° respectively. Its testing wave length is 240 nm and the scan range of wavelength in DAD testing device is 190–400 nm.

**Table 2:** Linear gradient elution program of mobile phase

Time/min	0.2% formic acid/water	0.1% formic acid/methanol
0-5	80-90%	10-20%
5-10	40-80%	20-60%
10-15	35-40%	60-65%
15-30	0-35%	65-100%
30-35	0%	100-100%

#### Preparation of standard solution

Precisely weigh a certain amount of contrast material of magnolol and hononkiol and put them into a volumetric flask, and then resolve them by adding methanol and consider it as the mixed standard solution.

#### Preparation of sample solution

Prepare and accurately weigh 0.2 g of medicine powder and put it into a 50 ml flask with added methanol for depositing to the right graduation. After this step, accurately weigh the samples and subject to ultrasonic extraction for 1 h, add the lost weight, and filter it through a 0.22 μm filtration membrane, and get the sample solution. Accurately weigh 1 μl of the sample solution for sample feeding and for the analysis of UPLC, and record the chromatogram of sample.

## RESULTS

### Optimization of extracting conditions

#### Extraction solvent

The pulverized samples were subjected to ultrasonic extraction for 1 h with 100% EtOH, 70% EtOH (v/v), MeOH and n-BuOH, respectively; more peaks were obtained in the extracts of methanol. According to the principle that more chemical composition should be retained to evaluate traditional Chinese medicine, methanol was finally chosen as the extraction solvent.

#### Extraction method

Using methanol as the extraction solvent, the pulverized samples were reflux extracted for 3 h, soxhlet extracted for 3 h, ultrasonic extracted twice for 1 h each, respectively. More peaks were obtained in the extract that was ultrasonic extracted for 1 h, and less time was consumed. Ultrasonic extraction for 1 h was finally chosen as the extraction method.

### The methodology examination of the fingerprint

#### Precision experiment

By taking the same sample solution and continually feed for six times, it is known that the similarity is higher than 0.97, and the RSD of the relative retention time and relative peak area of the common peaks are 0.70% and 2.6%, respectively, which shows a high accuracy.

#### The experiment of stability

By taking the same sample solution and feeding at different time 0, 4, 8, 12, 18, 24 h, we found that the similarities were all above 0.96 and the RSD of the relative retention time and relative peak area of the common peaks are 0.93% and 2.9% respectively, which shows a good stability.

#### The experiment of reproducibility

By dividing the same sample powder into six parts and each of 0.2 g with precision, we separately afflux the samples solution prepared according to the above method and got the result that the similarities of the chromatogram were greater than 0.96, and the RSD of the relative retention time and relative peak area of the common peaks are 0.92% and 2.2% respectively, which shows a good reproducibility.

### Communion mode validation of fingerprint

**Figure 1:**
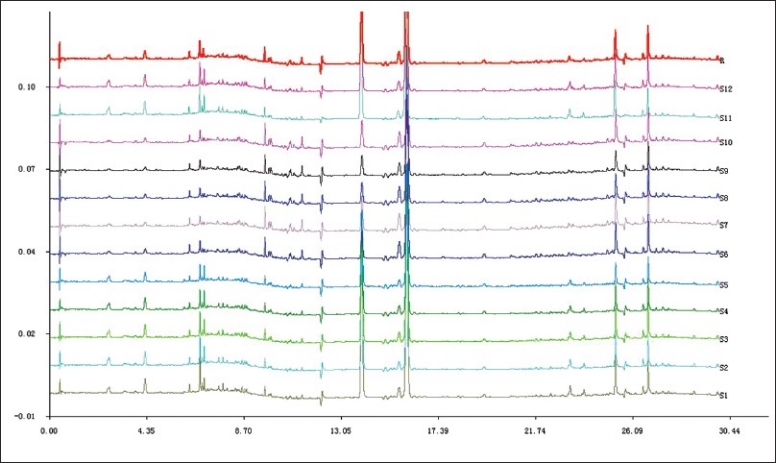
Chromatogram of the fingerprints of the twelve samples

The twelve sample solutions from two species in five areas in Zhejiang Province were prepared and analyzed by UPLC, and chromatograms of the samples were recorded at 240 nm. The chromatographic peaks of different samples with the same relative retention time were defined as the characteristic peaks. Forty-one peaks were identified as the characteristic peaks. Among the characteristic peaks, peak 22 was identified as magnolol contrasting with the magnolol standard, peak 18 was identified as hononkiol contrasting with the hononkiol standard. Chromatogram of the fingerprints of the samples can be seen in Figure 1.

The data of the chromatographic fingerprints were imported into the Similarity Evaluation System for Chromatographic Fingerprint of TCM (Version 2004 A) software.[12–14] In this study, a similarity analysis based on matching against the median of the fusion vectors of all the samples was performed. Reference chromatogram was generated by median method. The calculational results of relative peak area of communion peak in twelve samples are listed in Table 3, and those of similarity of the communion peak in twelve samples are listed in Table 4.

**Table 3:** The calculational result of relative peak area of communion peak

No.	Retention time	S1	S2	S3	S4	S5	S6	S7	S8	S9	S10	S11	S12
1	2.67	0.032	0.047	0.043	0.046	0.048	0.021	0.018	0.024	0.019	0.020	0.048	0.044
2	3.85	0.007	0.008	0.007	0.009	0.012	0.009	0.008	0.009	0.010	0.009	0.008	0.008
3	4.27	0.052	0.057	0.054	0.056	0.052	0.025	0.017	0.023	0.024	0.023	0.061	0.056
4	4.94	0.006	0.007	0.007	0.008	0.009	0.008	0.007	0.009	0.009	0.007	0.009	0.008
5	6.03	0.008	0.005	0.011	0.016	0.047	0.007	0.007	0.007	0.037	0.006	0.007	0.010
6	6.25	0.023	0.030	0.026	0.030	0.039	0.046	0.042	0.050	0.060	0.043	0.025	0.030
7	6.72	0.087	0.072	0.064	0.061	0.064	0.044	0.034	0.042	0.069	0.042	0.069	0.077
8	7.07	0.009	0.020	0.021	0.024	0.023	0.017	0.021	0.028	0.029	0.025	0.010	0.010
9	7.20	0.031	0.067	0.055	0.072	0.078	0.053	0.040	0.049	0.048	0.043	0.037	0.033
10	8.32	0.009	0.071	0.061	0.066	0.071	0.039	0.034	0.041	0.042	0.038	0.007	0.007
11	8.46	0.012	0.035	0.036	0.040	0.044	0.042	0.039	0.047	0.048	0.043	0.012	0.014
12	8.60	0.013	0.026	0.024	0.025	0.029	0.021	0.018	0.023	0.023	0.021	0.013	0.012
13	9.63	0.018	0.022	0.026	0.026	0.028	0.046	0.048	0.054	0.056	0.052	0.024	0.027
14	9.89	0.014	0.018	0.022	0.023	0.023	0.022	0.025	0.028	0.031	0.026	0.018	0.021
15	10.77	0.014	0.021	0.023	0.026	0.040	0.063	0.064	0.074	0.072	0.068	0.014	0.022
16	11.28	0.012	0.013	0.018	0.019	0.020	0.129	0.121	0.149	0.173	0.132	0.011	0.016
17	12.20	0.057	0.124	0.073	0.085	0.104	0.084	0.086	0.109	0.121	0.096	0.028	0.061
18	13.96	0.618	0.616	0.545	0.515	0.483	0.131	0.155	0.121	0.148	0.134	0.616	0.559
19	15.02	0.015	0.019	0.019	0.022	0.026	0.022	0.020	0.026	0.028	0.023	0.008	0.016
20	15.41	0.018	0.023	0.021	0.028	0.032	0.025	0.022	0.030	0.034	0.026	0.003	0.014
21	15.63	0.060	0.062	0.073	0.075	0.073	0.114	0.103	0.128	0.109	0.105	0.051	0.067
22	15.97	1.000	1.000	1.000	1.000	1.000	1.000	1.000	1.000	1.000	1.000	1.000	1.000
23	16.30	0.019	0.024	0.023	0.028	0.032	0.019	0.017	0.022	0.024	0.019	0.008	0.010
24	19.43	0.018	0.022	0.021	0.025	0.026	0.028	0.026	0.033	0.020	0.021	0.014	0.021
25	20.63	0.011	0.012	0.013	0.015	0.016	0.019	0.019	0.022	0.021	0.019	0.009	0.011
26	21.09	0.009	0.010	0.011	0.007	0.013	0.010	0.011	0.012	0.011	0.009	0.010	0.009
27	21.76	0.008	0.009	0.010	0.008	0.008	0.015	0.015	0.018	0.017	0.015	0.009	0.014
28	21.97	0.005	0.006	0.006	0.008	0.007	0.018	0.018	0.020	0.021	0.018	0.005	0.015
29	22.41	0.007	0.009	0.008	0.009	0.008	0.006	0.006	0.007	0.007	0.006	0.009	0.008
30	23.26	0.043	0.047	0.045	0.046	0.042	0.017	0.016	0.025	0.026	0.016	0.051	0.050
31	23.90	0.027	0.030	0.027	0.028	0.019	0.008	0.014	0.009	0.008	0.010	0.027	0.024
32	24.94	0.014	0.018	0.020	0.024	0.007	0.038	0.035	0.019	0.035	0.039	0.006	0.005
33	25.31	0.177	0.208	0.213	0.227	0.213	0.235	0.213	0.129	0.238	0.241	0.141	0.173
34	25.76	0.071	0.089	0.096	0.112	0.077	0.053	0.084	0.106	0.090	0.081	0.013	0.023
35	26.55	0.046	0.056	0.062	0.074	0.022	0.052	0.064	0.072	0.022	0.023	0.023	0.025
36	26.77	0.142	0.168	0.187	0.205	0.131	0.184	0.212	0.251	0.138	0.143	0.099	0.120
37	27.13	0.020	0.027	0.032	0.038	0.015	0.026	0.041	0.056	0.021	0.019	0.007	0.008
38	27.43	0.055	0.069	0.074	0.082	0.038	0.027	0.055	0.080	0.045	0.027	0.008	0.010
39	27.68	0.032	0.034	0.038	0.050	0.060	0.013	0.052	0.080	0.034	0.027	0.008	0.008
40	28.83	0.010	0.012	0.011	0.011	0.060	0.005	0.008	0.056	0.094	0.007	0.008	0.008
41	29.88	0.010	0.016	0.017	0.018	0.020	0.018	0.018	0.020	0.024	0.020	0.016	0.015

**Table 4:** The calculational result of similarity of communion peak

	S1	S2	S3	S4	S5	S6	S7	S8	S9	S10	S11	S12	Reference
S1	1.000	0.971	0.993	0.984	0.979	0.878	0.890	0.857	0.864	0.878	0.990	0.991	0.957
S2	0.971	1.000	0.974	0.970	0.965	0.860	0.873	0.846	0.853	0.860	0.956	0.960	0.937
S3	0.993	0.974	1.000	0.994	0.985	0.902	0.915	0.888	0.885	0.901	0.976	0.981	0.970
S4	0.984	0.970	0.994	1.000	0.985	0.908	0.922	0.896	0.895	0.907	0.967	0.976	0.974
S5	0.979	0.965	0.985	0.985	1.000	0.906	0.911	0.881	0.905	0.907	0.968	0.977	0.972
S6	0.878	0.860	0.902	0.908	0.906	1.000	0.994	0.970	0.976	0.995	0.869	0.892	0.959
S7	0.890	0.873	0.915	0.922	0.911	0.994	1.000	0.984	0.972	0.993	0.874	0.897	0.963
S8	0.857	0.846	0.888	0.896	0.881	0.970	0.984	1.000	0.960	0.969	0.835	0.858	0.935
S9	0.864	0.853	0.885	0.895	0.905	0.976	0.972	0.960	1.000	0.981	0.853	0.878	0.939
S10	0.878	0.860	0.901	0.907	0.907	0.995	0.993	0.969	0.981	1.000	0.868	0.892	0.958
S11	0.990	0.956	0.976	0.967	0.968	0.869	0.874	0.835	0.853	0.868	1.000	0.997	0.949
S12	0.991	0.960	0.981	0.976	0.977	0.892	0.897	0.858	0.878	0.892	0.997	1.000	0.965
Reference	0.957	0.937	0.970	0.974	0.972	0.959	0.963	0.935	0.939	0.958	0.949	0.965	1.000

## CONCLUSION

This experiment established the use of UPLC fingerprint for the identification of *Magnoliae officinalis* cortex processed with ginger juice collected from two species in five areas in Zhejiang Province. Twelve samples were analyzed by this method, and the chromatographic data were processed by similarity analysis. The experiment result shows that the similarities of all the samples were greater than 0.93 through analysis of the fingerprint similarity in samples of 12 different species and regions. Chromatographic profile of different samples was consistent. The holistic separation extent is better, having a total of 41 communion peaks, with their own chemical barcode features, setting up a communion of more reliable models. The experimental results show the intrinsic species and quantities of the chemical composition.

Compared with the reference chromatogram generated by median method, the similarity of the 12 different species of *Magnoliae officinalis* cortex processed samples produced in Zhejiang is above 0.93. This shows that there is a high similarity in the 12 different *Magnoliae officinalis* cortex processed samples. From this result, we can conclude preliminarily that the 12 different species of *Magnoliae officinalis* cortex processed sample produced in Zhejiang are quite similar, with stable quality. And the result also shows that the processed method used in this experiment is reliable and stable. Therefore, its feasible to use UPLC fingerprints technology as the quality controlling means for *Magnoliae officinalis* cortex processed samples. Overall, from the above results, we can conclude that the main components of *Magnoliae officinalis* cortex processed samples are the 41 communion peaks in the chromatogram. In practice, when we want to control the quality of the traditional Chinese medicinal materials, we should compare the chromatogram of the testing medicine herbs with the chromatogram generated by the median method. If the similarity of the testing traditional Chinese medicinal materials is greater than 0.90, it shows that these medicine herbs have reliable quality and source. If its similarity is less than 0.90, it shows that there are some problems in its source and quality, so we must be careful in using it.

The UPLC fingerprint technology established by this experiment has the characteristic of high speed, accuracy and high efficiency, and it can be used as an effective means of appraising and identifying the quality of *Magnoliae officinalis* cortex processed.
